# Case Report Reconstruction of Exposed Ilium With Reverse Turnover Latissimus Dorsi Muscle Flap

**Published:** 2011-04-14

**Authors:** Kenji Hayashida, Yoshie Endo, Katsuhiko Kamebuchi

**Affiliations:** Department of Plastic Surgery, Oita Nakamura Hospital, Oita-shi, Oita, Japan

## Abstract

**Objective:** It is difficult to cover a large skin and soft tissue defect with exposure of the ilium. We therefore performed a new reconstruction technique, using a reverse latissimus dorsi muscle flap fed by perforating branches of only the 10th intercostal artery. **Methods:** A 45-year-old man had a large traumatic defect located on the hip with exposure of the iliac crest. After confirming and preserving perforating branches of the 10th intercostal artery, the latissimus dorsi muscle flap was turned over just proximal to the perforating branch, and a split-thickness skin graft was performed over the flap. **Results:** The skin graft took place well and there were no circulation problems. **Conclusions:** This flap covered a larger area on the hip than the musculocutaneous flap. Furthermore, this is easier to perform and is less invasive than a vascularized free flap. Skin and soft tissue defects that expose bones of the lumbar or hip region can be reconstructed with a local flap; however, the deficit is small for this coverage and usually there is little skin and soft tissue to cover the wound defect in the surrounding area. Thus, it is often difficult to deal with large defects. We performed a reconstruction, using a reverse latissimus dorsi flap fed by perforating branches of the 10th intercostal artery for a large skin and soft tissue defect of the hip with exposure of the iliac crest, resulting in a good outcome. This technique is thought to be useful for reconstruction when the ilium is exposed, and we report the case and surgical procedure.

## CASE REPORT

### Patient: A 45-year-old man

The patient was in a severe shock, with systolic blood pressure in the 60s (mm Hg). Skin and soft tissue necrosis was seen on the right leg, accompanied by a foul odor. Clear inflammation was seen, with white blood cells count 17 600 (/µL) and C-reactive protein 27 (mg/dL). Right leg radiography findings showed a subcutaneous gas pattern over the entire circumference of the leg from the right foot to the right inguinal region. The patient was admitted and underwent surgery with a diagnosis of impaired consciousness from sepsis caused by gas gangrene of the right leg. The results of a final bacterial culture of the wound showed *Escherichia coli* and this was the probable candidate for the causative pathogen.

Preserving the right leg was, therefore, considered impossible and transfemoral amputation and debridement of the lower abdomen were performed. The wound was left open, creating a large skin and soft tissue defect from the right hip to the right femoral stump that exposed the entire right iliac crest.

**Figure 1:**
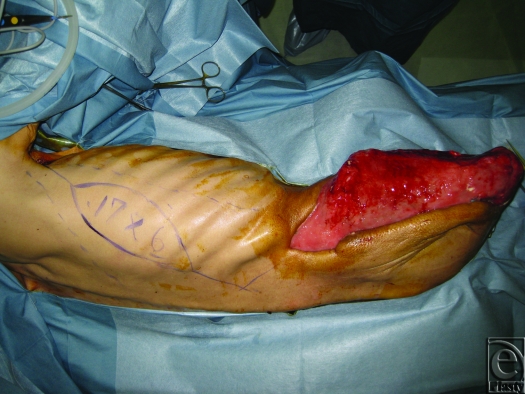

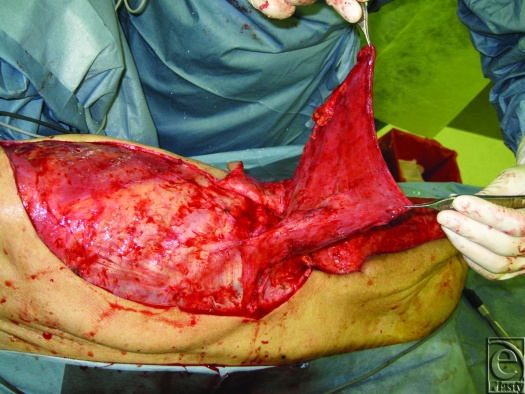

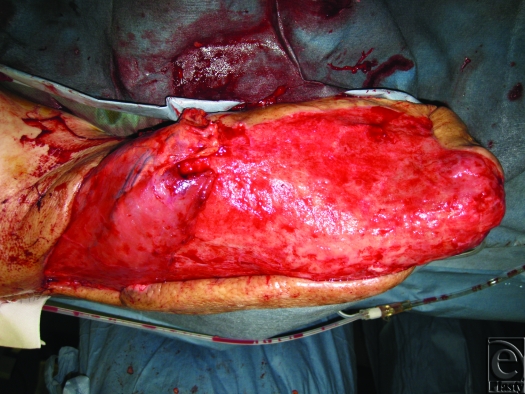
(A) Intraoperative findings. Large skin and soft tissue defect that exposes the right iliac crest. (B) Intraoperative findings. Elevation of the reverse latissimus dorsi flap. (C) Intraoperative findings. Iliac crest covered with the muscle flap.

**Figure 2:**
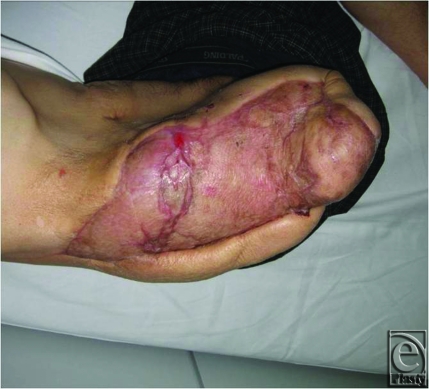
Three months postoperatively. No skin ulcers are seen.

Reconstruction was performed about 3 weeks after the initial surgery (Fig 1A–1C). Coverage with a local flap was thought to be impossible since the entire right iliac crest was exposed, and initial reconstruction with a reverse latissimus dorsi flap was planned to cover the exposed area. We, therefore, situated perforators of the 10th intercostal artery by Doppler before the surgery. During the surgery, when the latissimus dorsi flap was explored and elevated, the main nutrient vessels of the thoracodorsal artery and veins were transiently clamped and only blood flow from the 10th intercostals vessels was confirmed. The right latissimus dorsi was detached near the humerus terminal area and retrogradely elevated, but the area that could be reached by flap rotation was limited and the exposed area could not be covered; therefore, the skin was cut from the musculocutaneous flap and the muscle flap was used. After confirming and preserving perforating branches of the 10th intercostal artery, the muscle flap was turned over just proximal to the perforating branch. Detachment of the perforating branch and muscle flap was continued until the entire exposed iliac crest up to and including the superior anterior iliac spine could be covered. A split-thickness skin graft was performed from the buttocks for the turnover latissimus dorsi and soft tissue exposure, and the surgery was completed. Most of the skin graft took place, and 6 weeks after reconstruction surgery the patient in a wheelchair left the hospital (Fig 2).

## DISCUSSION

Reverse latissimus dorsi flaps are useful for covering skin and soft tissue defects of the back, such as those from meningomyelocele or pressure ulcer, and their application in such cases has been reported^1^,^2^; however, they have disadvantages including a thickened pedicle in the musculocutaneous flap and the limited arc of the flap. Therefore, the perforating branches of the intercostal arteries can be preserved in the latissimus dorsi flap and the musculocutaneous flap is trimmed to a muscle flap, so the area reached can be extended by “turning over” the flap. Applying this method, we further transected the perforating branch of the 9th intercostal artery and, after confirming the perforating branch of the 10th intercostal artery, turned over the muscle flap just proximal to that perforating branch. We continued to detach the perforating branch and latissimus dorsi so that the area covered by the latissimus dorsi reached the superior anterior iliac spine. The upper iliac crest can be reached by simply rotating and transferring the muscle flap alone, but with the present method it was possible to reach the superior anterior iliac spine by turning over and transferring the muscle flap. This enables reaching a distance about 10 cm farther than with rotation. The skin graft took place well and there were no tissue circulation problems.

Some past reports have described supercharging the thoracodorsal artery and superior gluteal artery after elevating the reverse latissimus dorsi flap,^3^ and others state that the procedure is safer if it is performed after a delay.^4^ We were also prepared to supercharge the thoracodorsal artery to the inferior epigastric artery or another artery during surgery if circulation was unstable, but bleeding from part of the humerus terminal area, which is the most distal portion of the latissimus dorsi muscle, was observed. We therefore considered that supercharging or other additional circulation to the reverse latissimus dorsi flap was not necessary even if the perforating branch of the ninth intercostal artery was transected. Watanabe et al^5^ reported a useful study that provides support for these results. In a 3-dimensional anatomic study using angiography, they observed 3 vascular territories anatomically in the latissimus dorsi and reported that the perforating branch of the 10th intercostal artery was directly connected to all 3 territories via choke vessels. We therefore concluded that it is possible to supply nutrients to the entire latissimus dorsi if only the perforating branch of the 10th intercostal artery is included as a nutrient vessel.

The present method uses a muscle flap, so it requires additional skin grafting; however, because the muscle flap is elevated with the patient in the lateral position, a graft can be harvested from the buttocks or back without changing the body position and the surgery can be concluded in the same position quickly.

Other methods proposed for reconstructive surgery of an exposed ilium with a large skin and soft tissue defect include pedicled rectus abdominis musculocutaneous flaps and free flaps. In the present case, we predicted that the patient would be in a wheelchair after the surgery, and if we had used a rectus abdominis muscle, the risk of abdominal hernia would have been high. We therefore did not use a rectus abdominis flap for reconstruction. Although the area of detachment with the present method is fairly large, the procedure is simple and little is sacrificed. As a result, there are almost no limitations in daily life following surgery. It is difficult to accurately confirm the position of the perforating branch of the intercostal artery preoperatively with a Doppler blood flow meter, but the perforating branch can be seen in detail with the use of multislice detector computed tomography. This will probably enable faster and more reliable elevation. The present method is thought to be applicable in cases of iliac pressure ulcer or osteomyelitis of the ilium. It is, therefore, a useful method for reconstruction of ilium exposure.

## CONCLUSION

We have reported reconstruction with a reverse latissimus dorsi flap for a skin and soft tissue defect of the hip with exposure of the ilium. The procedure is simple and the blood circulation is stable, and it is thought to be an effective reconstruction method for ilium exposure.

## References

[1] Muramatsu K, Ihara K, Ooi R, Imazyo Y (2006). Experiences with the “reverse” latissimus dorsi myocutaneous flap. Plast. Reconstr Surg.

[2] Yamamoto N, Igota S, Izaki H (2001). “Reverse turnover” transfer of a latissimus dorsi muscle flap to a large lumbar defect. Plast Reconstr Surg.

[3] Giesswein P, Constance CG, Mackay DR (1994). Supercharged latissimus dorsi muscle flap for coverage of the problem wound in the lower back. Plast Reconstr Surg.

[4] Camus E, Martinot-Duquennoy E, Patenotre P (1996). Surgical cover of thoracolumbar radiation necrosis, report of six cases. Ann Chir Plast Esthet.

[5] Watanabe K, Kiyokawa K, Rikimaru H (2010). Anatomical study of latissimus dorsi musculocutaneous flap vascular distribution. J Plast Reconstr Aesthet Surg.

